# Barreras para el acceso a la atención integral de las personas afectadas por la coinfección por tuberculosis y virus de inmunodeficiencia humana en Perú, 2010–2015

**DOI:** 10.26633/RPSP.2017.23

**Published:** 2017-04-21

**Authors:** Lisset García-Fernández, Carlos Benites, Byelca Huamán

**Affiliations:** 1 Estrategia Sanitaria Nacional de Infecciones de Transmisión Sexual VIH/SIDA Estrategia Sanitaria Nacional de Infecciones de Transmisión Sexual VIH/SIDA Perú Estrategia Sanitaria Nacional de Infecciones de Transmisión Sexual VIH/SIDA. Ministerio de Salud de Perú, Perú.

**Keywords:** Coinfección, tuberculosis, VIH, Perú, Coinfection, tuberculosis, HIV, Peru, Coinfecção, tuberculose, HIV, Peru

## Abstract

**Objetivo.:**

*Identificar las barreras programáticas que dificultan el acceso a la atención integral de pacientes con coinfección por tuberculosis y virus de la inmunodeficiencia humana (TB/VIH)*.

**Métodos.:**

*Se trata de un estudio de métodos mixtos. La investigación cualitativa se realizó mediante entrevistas en profundidad a actores clave y el componente cuantitativo a través del análisis descriptivo de corte transversal de datos programáticos del período 2010–2015 sobre los programas de tuberculosis y VIH de establecimientos de salud de las ciudades de Lima e Iquitos*.

**Resultados.:**

*Se entrevistaron a 22 actores clave en siete establecimientos. Las barreras identificadas fueron: poca o ninguna coordinación entre los equipos de tuberculosis y VIH, manejo por separado de los casos de tuberculosis y de VIH en diferentes niveles de atención, financiamiento insuficiente, recursos humanos escasos o poco capacitados y ausencia de un sistema de información integrado. Se evidenció que el tamizaje para VIH en pacientes con tuberculosis se incrementó (de 18,8% en 2011 a 95,2% en 2015), la cobertura de isoniazida en pacientes con VIH disminuyó (de 62% a 9%) y la proporción de fallecidos entre los casos de coinfección por TB/VIH fue de 20% en promedio*.

**Conclusiones.:**

*Existe una débil coordinación entre las estrategias sanitarias sobre VIH y sobre tuberculosis. El manejo de la coinfección por TB/VIH es fragmentado en diferentes niveles de atención, lo que repercute en la atención integral del paciente. Como producto de esta investigación, se elaboró un documento técnico para establecer los procedimientos conjuntos, el cual deberá ser implementado para una mejora en la atención integral de la coinfección por TB/VIH*.

## Abstract (en)

**Objective.:**

Identify the programmatic barriers that hinder access to comprehensive care of patients with tuberculosis and human immunodeficiency virus (TB/HIV) coinfection.

**Methods.:**

This is a mixed-method study. Qualitative research was conducted via indepth interviews with key actors and the quantitative component involved cross-sectional descriptive analysis of programmatic data from 2010-2015 on tuberculosis and HIV programs at health facilities in the cities of Lima and Iquitos.

**Results.:**

Twenty-two key actors in seven establishments were interviewed. The identified barriers were: little or no coordination between tuberculosis and HIV teams, separate management of tuberculosis and HIV cases at different levels of care, insufficient financing, limited or poorly trained human resources, and lack of an integrated information system. It was found that HIV screening in TB patients increased (from 18.8% in 2011 to 95.2% in 2015), isoniazid coverage of HIV patients declined (from 62% to 9%), and the proportion of deaths among TB/HIV coinfection cases averaged 20%.

**Conclusions.:**

There is poor coordination between HIV and TB health strategies. Management of TB/HIV coinfection is fragmented into different levels of care, which has an impact on comprehensive patient care. As a result of this research, a technical document was prepared to establish joint procedures that should be implemented to improve comprehensive care of TB/HIV coinfection.

## RESUMEN

**Objetivo.:**

Identificar as barreiras programáticas que dificultam o acesso à atenção integral de pacientes com coinfecção por tuberculose e vírus da imunodeficiência humana (TB/HIV).

**Métodos.:**

Estudo de métodos mistos. A pesquisa qualitativa foi realizada mediante entrevistas aprofundadas com atores-chave, e o componente quantitativo baseou-se na análise descritiva transversal de dados programáticos dos programas de tuberculose e HIV de estabelecimentos de saúde das cidades de Lima e Iquitos no período de 2010 a 2015.

**Resultados.:**

Foram entrevistados 22 atores-chave em sete estabelecimentos. As barreiras identificadas foram: pouca ou nenhuma coordenação entre as equipes de tuberculose e HIV, manejo separado dos casos de tuberculose e HIV em diferentes níveis de atenção, financiamento insuficiente, recursos humanos escassos ou pouco capacitados e ausência de um sistema de informação integrado. Constatou-se que o rastreamento de HIV em pacientes com tuberculose aumentou (de 18,8% em 2011 para 95,2% em 2015), a cobertura da profilaxia com isoniazida em pacientes com HIV diminuiu (de 62% para 9%) e a proporção média de óbitos entre os casos de coinfecção por TB/HIV foi de 20%.

**Conclusões.:**

Existe uma má coordenação entre as estratégias de saúde para HIV e tuberculose. O manejo da coinfecção por TB/HIV é fragmentada nos diferentes níveis de atenção, o que prejudica a atenção integral do paciente. Esta pesquisa resultou na elaboração de um documento técnico para estabelecer os procedimentos conjuntos que deverão ser implementados para melhorar a atenção integral da coinfecção por TB/HIV.

Las personas infectadas por el virus de la inmunodeficiencia humana (VIH) tienen 29 veces más probabilidades de desarrollar tuberculosis que las personas que no tienen VIH (1). A nivel mundial, la incidencia de tuberculosis en personas que viven con VIH (PVV) es de 105,2 por cada 100 000 habitantes y en la región andina de América Latina es de 118,4 por cada 100 000 habitantes (2). Por otro lado, la incidencia de tuberculosis en personas sin VIH a nivel mundial es de 98,7 por cada 100 000 habitantes (2), mientras que en la región andina de América Latina es de 117,9 por cada 100 000 habitantes. Asimismo, el riesgo de tuberculosis en las PVV que no reciben tratamiento antirretroviral (TARV) es nueve veces mayor que los pacientes que sí reciben tratamiento (2). Esta diferencia es incluso 15 veces mayor en los individuos que tienen recuentos de CD4 menores de 200 células/mL (2), de lo cual surge la necesidad de que todos los pacientes con sida, en especial aquellos con recuentos de CD4 menores de 200, reciban profilaxis con isoniazida, tal como se recomienda en diferentes guías de manejo (3–5).

En el nivel global, en 2013 hubo 1,1 millones de personas con coinfección por tuberculosis/VIH (TB/VIH) (1). Las muertes por tuberculosis en personas con VIH han disminuido de 540 000 en 2004 a 360 000 en 2013 (6). Sin embargo, la tuberculosis sigue siendo la causa más importante de muerte en PVV en el mundo (6).

En el Perú, para el año 2014, se reportaron 1 094 casos de coinfección por TB/VIH (7). En total, la proporción de coinfección por TB/VIH es alrededor del 3% (8). Asimismo, las epidemias de tuberculosis y VIH comparten su distribución epidemiológica (8). Según datos programáticos, el VIH y la tuberculosis se concentran principalmente en las zonas urbanas de la costa y la selva, y afectan sobre todo a los hombres en la población económicamente activa (7, 9). Así, la proporción de coinfección por TB/VIH es mayor en las regiones de Loreto (6,4%), Callao (6,3%) y Lima Norte (5%) (8).

Con el objetivo de reducir el problema de la coinfección por TB/VIH, desde el año 2004, la Organización Mundial de la Salud (OMS) (10) y otras organizaciones internacionales (11) han recomendado la aplicación de actividades de colaboración sobre TB/VIH. Las recomendaciones de la OMS se centran en la implementación (o fortalecimiento) de los servicios integrados para la coinfección por TB/VIH, la reducción de la carga de enfermedad TB/VIH y el TARV temprano (10). Con esto, se han evidenciado mejoras a nivel mundial, como el incremento del acceso al TARV de los pacientes con coinfección por TB/VIH de 47%, en el 2012, a 65% para el 2013 (6). En América Latina, la cobertura de TARV fue de 76%; sin embargo, en los países de esta región las actividades de colaboración entre los programas de tuberculosis y VIH han presentado dificultades como la falta de políticas nacionales conjuntas y la ausencia de actividades de integración en el nivel operativo (12).

El Ministerio de Salud del Perú, a través de las Estrategias Nacionales de Salud para la tuberculosis y el VIH/sida, es el encargado de la prevención y control de estos problemas de salud. Así, la Norma Nacional de Tuberculosis (13) y la Norma Nacional de VIH (14), delinean las intervenciones que deberían ser realizadas para cada una de estas enfermedades. Sin embargo, los reportes muestran bajas coberturas en los indicadores de la coinfección, lo cual sugiere dificultades en la implementación y articulación para la atención de los pacientes con coinfección por TB/VIH por parte de ambos programas (conocidos en el Perú como Estrategias).

Se sabe que una de las principales dificultades para la atención de los pacientes con coinfección por TB/VIH es que esta se realiza por separado para tuberculosis y para VIH. Incluso, en la mayor parte de casos, los pacientes con coinfección por TB/VIH reciben tratamiento para tuberculosis en un establecimiento de salud del primer nivel de atención y el TARV en otro establecimiento, en general del segundo o tercer nivel de atención. Se desconoce en detalle cuáles son los elementos que impiden que los equipos de las Estrategias Sanitarias de Tuberculosis y VIH coordinen acciones destinadas al mejor cuidado de los pacientes con coinfección por TB/VIH.

El objetivo de esta investigación fue identificar las barreras programáticas para la atención integral de los pacientes con coinfección por TB/VIH. Se recogió información de los servicios que brindan atención a pacientes con tuberculosis y VIH, para identificar las brechas programáticas de la atención de la coinfección por TB/VIH, y que sirva para la elaboración e implementación de una política nacional conjunta que aborde la problemática sobre TB/VIH.

## MATERIALES Y MÉTODOS

**FIGURA 1.**
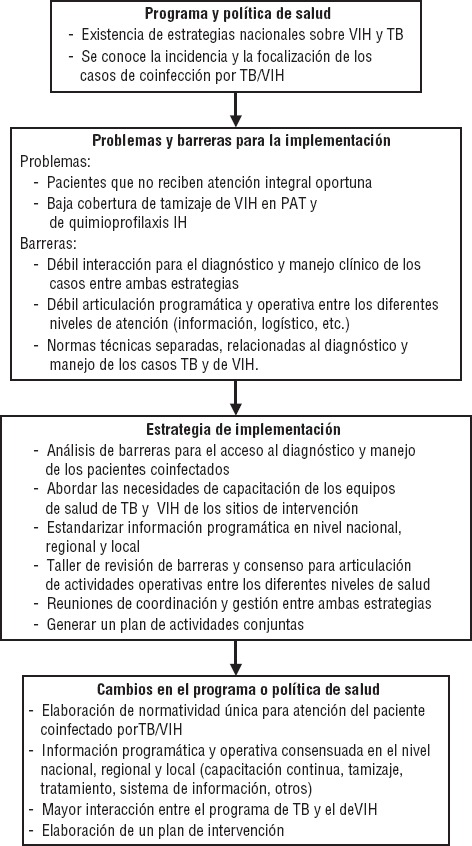
Diagrama de flujo del desarrollo de la investigación

VIH, virus de la inmunodeficiencia humana; TB, tuberculosis; IH, isoniazida; PAT, personas afectadas por la tuberculosis.

Este trabajo forma parte de una nueva iniciativa: “Mejoras en la ejecución de programas a través de investigaciones integradas en los mismos acerca de su ejecución (iPIER)”, desarrollado por la Alianza para la Investigación en Políticas y Sistemas de Salud (AHPSR), en colaboración con la Organización Panamericana de la Salud (OPS). El modelo iPIER jerarquiza a los ejecutores de programas como agentes clave de investigación con el objetivo de entender las fallas en los sistemas de salud que crean barreras a la implementación, así como permite identificar las soluciones a estas barreras. La investigación sobre la ejecución de programas integrada en los procesos existentes apoyan su efectividad y políticas de salud eficaces a través de la utilización de la investigación que se llevó a cabo como parte del proceso de implementación (figura 1). Una descripción detallada de la aplicación de la metodología de investigación se incluye en el documento conceptual iPIER “Barreras para el acceso oportuno a la atención integral de las personas afectadas por la coinfección TB/VIH en el Perú”.

**FIGURA 2.**
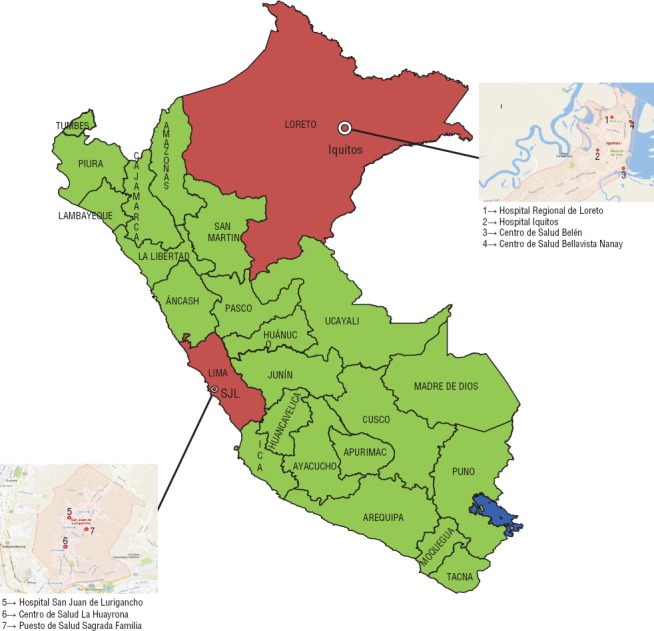
Ubicación de los establecimientos de salud incluidos en el estudio

Se llevó a cabo un estudio aplicando métodos mixtos, que constó de un componente cualitativo para la identificación de las barreras en la atención de los pacientes con coinfección por TB/VIH, y un componente cuantitativo para identificar las brechas programáticas. El estudio se realizó en Lima e Iquitos, ciudades con alta incidencia de tuberculosis y VIH (8). Se seleccionaron siete establecimientos de salud ubicados en el distrito de San Juan de Lurigancho (Lima) y en tres distritos de la ciudad de Iquitos (figura 2), que se eligieron por contar con la mayor cantidad de pacientes con tuberculosis o VIH. Las características de los establecimientos se describen en el cuadro 1.

El componente cualitativo se realizó mediante entrevistas en profundidad a actores clave en el proceso de la atención. Se seleccionaron a los coordinadores de las estrategias de tuberculosis y VIH de los establecimientos de salud, médicos, enfermeras, obstetras y a personas con coinfección por TB/VIH. Además, se entrevistó a profesionales que trabajan en los equipos técnicos de las estrategias sanitarias del nivel nacional. Se utilizó un cuestionario semiestructurado administrado por un encuestador, diferenciado para los proveedores de salud y para los pacientes. Todas las entrevistas se grabaron utilizando un dispositivo de audio y luego fueron transcritas. El análisis se realizó mediante un proceso iterativo de lectura de las transcripciones, selección de temas y codificación; se utilizó el *software* ATLAS.ti versión 7.2^®^.

Para el componente cuantitativo se inspeccionaron los datos programáticos de las estrategias de tuberculosis y VIH en los establecimientos de salud. Se consultaron los registros administrativos y clínicos existentes para los años 2010 a 2015 de cada establecimiento de salud. Además, se solicitó información adicional al personal de salud para complementar. El instrumento utilizado para la recolección de datos fue elaborado adaptando la lista de verificación de un proyecto similar realizado en Honduras (15). El instrumento constó de las siguientes secciones: datos de identificación, aspectos generales, atención de la tuberculosis y el VIH y datos para la construcción de indicadores programáticos. La información se analizó utilizando la estadística descriptiva, en hoja de cálculo Excel versión 2010^®^.

El estudio contó con la aprobación del Comité de Ética del Hospital Nacional Hipólito Unanue (Ref. N° 059-2015-CIEI-HNHU) y del Comité de Revisión Ética de la Organización Panamericana de la Salud -PAHOERC- (Ref. N° PAHO-2015-03-0015). Previo a la recolección de la información se realizó la presentación del objetivo y procedimientos del estudio al jefe o encargado en cada establecimiento de salud. Posteriormente, se invitó a participar del estudio al personal de salud de las estrategias de tuberculosis y VIH del establecimiento. Los pacientes fueron invitados a través del personal de salud. Para participar en el estudio se realizó la lectura y firma del consentimiento informado.

## RESULTADOS

### Componente cualitativo

Se entrevistó a 22 actores clave (16 proveedores de salud, cuatro pacientes y dos funcionarios). Se identificaron varias barreras en la atención integral de paciente con TB/VIH.

La primera barrera es la poca o ninguna coordinación de las actividades, como el tamizaje para VIH en pacientes con tuberculosis o seguimiento de la adherencia a los tratamientos. La experiencia y conocimientos del personal de salud influyen en este relacionamiento. Otros factores son la voluntad e interés del personal, la cercanía física y la afinidad entre los responsables.

“*Bueno, con las compañeras [de VIH] no sé qué actividades harán, en eso reconozco que estamos distanciadas. Creo que sería cuestión de que nosotros facilitemos la información porque el hospital no sabe qué pacientes hay en los otros centros […].”* (IQT_02_Ic)

La atención integral de la coinfección por TB/VIH implica que los servicios destinados al manejo de ambas patologías mantengan una comunicación fluida, de manera que se conozca el estado de cada paciente. Los principales resultados de esta falta de coordinación son la falta de vinculación a los servicios, como el caso los tamizados reactivos para VIH en los servicios de TB que no acuden a los servicios de VIH, o la pérdida del seguimiento de los pacientes, en el caso de los pacientes con VIH que fueron diagnosticados de TB y que no acuden al tratamiento.

En el Perú, el tratamiento de la coinfección por TB/VIH suele realizarse en diferentes establecimientos de salud, lo que dificulta la coordinación entre los equipos. Esto se debe a que los modelos de atención de la TB y el VIH difieren en varios sentidos. En el caso de la TB, los pacientes son atendidos en establecimientos de salud del primer nivel, cercanos a su domicilio, en los cuales reciben tratamiento directamente observado (DOT por sus siglas en inglés). Por otro lado, el manejo del VIH está implementado en algunos establecimientos de salud del segundo o tercer nivel, que cuentan con profesionales capacitados y los medicamentos se entregan en forma mensual o bimestral, según las necesidades de control del paciente, Esta fragmentación ocasiona desconocimiento del estado del paciente por parte de los profesionales, y la falta de adherencia a los tratamientos y problemas financieros para los pacientes.

“*[...] cerca de la casa hay una posta, el mismo neumólogo que me ve acá [en el hospital] me ve allá [...] porque acá me sale más caro. Acá solo vengo una vez al mes para recibir mis antirretrovirales.”* (H_IV)

Esta falta de coordinación se observa también a nivel programático, debido a la ausencia de un documento normativo que estandarice procedimientos y responsabilidades de cada estrategia sobre el manejo de la coinfección por TB/VIH, lo cual origina incertidumbre en el personal de salud.

“*Hay pacientes que vienen solos pero yo he adoptado estrategias con las que salgo de la norma o de la parte legal [...]. Yo misma hago la referencia para que el médico la firme, pero en realidad no consta que está haciendo referencia, pero eso me ha servido para captar más o hacer el tratamiento más oportuno [...]”*. (IQT_02_Ic)

Por otra parte, la falta de un sistema de información integrado dificulta el seguimiento de los pacientes coinfectados y el análisis de los datos en conjunto. Además, el registro se realiza en forma manual, lo que puede ocasionar pérdida de la información. Contribuye a este problema la ausencia de equipos informáticos y una red de internet que funcione de manera adecuada.

“*Solamente manejamos expedientes y las historias, no hay un sistema, me gustaría que esté interconectado pero no es así [...] al final acabamos nuevamente en lo empírico, todo manual y paloteando a fin de mes”*. (IQT_01_Ib)

Esta barrera se evidencia sobre todo a nivel central, ya que al momento de consolidar la información se obtienen datos inconsistentes. A su vez, es un reflejo de la falta de coordinación entre los servicios y el hecho que un mismo paciente acuda a establecimientos de salud distintos, en los cuales se utilizan diferentes identificadores, lo cual genera duplicación u omisión de la notificación.

**CUADRO 1.** Características de los establecimientos de salud visitados

Establecimiento de salud	Ciudad	Distrito	Nivel	Estrategia TB		Estrategia VIH	
				Baciloscopia	DOT	Tamizaje	TARV
Hospital Regional de Loreto “Felipe Santiago Arriola Iglesias”	Iquitos	Punchana	III-1	Sí	Restringido^a^	ELISA	Sí
Hospital Iquitos “César Garayar García”	Iquitos	Iquitos	II-2	Sí	Sí	Prueba rápida	Sí
Centro de Salud Belén de Villa Belén	Iquitos	Belén	I-3	Sí	Sí	Prueba rápida	No
Centro de Salud Bellavista Nanay	Iquitos	Punchana	I-4	Sí	Sí	Prueba rápida	No
Hospital San Juan de Lurigancho	Lima	SJL	II-2	Sí	Restringido^b^	ELISA	En proceso de acreditación
Centro de Salud La Huayrona	Lima	SJL	I-3	Sí	Sí	Prueba rápida	No
Puesto de Salud Sagrada Familia	Lima	SJL	I-2	Solo recolección de muestra	Sí	Prueba rápida	No

DOT, tratamiento en boca directamente observado (por sus siglas en inglés); TARV, tratamiento antirretroviral; TB, tuberculosis; VIH, virus de la inmunodeficiencia humana; ELISA, ensayo por inmunoadsorción ligado a enzimas (por sus siglas en inglés).

a Solo para pacientes de las inmediaciones y hospitalizados. Los diagnosticados son referidos a centros del primer nivel de atención.

b Solo para pacientes hospitalizados. Los diagnosticados son referidos a centros del primer nivel de atención.

Existen, además, barreras relacionadas con problemas en el sistema de salud, como el insuficiente financiamiento de las estrategias y la falta de recursos humanos. El personal entrevistado coincide en señalar que el tema de fondo no es la falta de dinero, sino la calidad del gasto. En general, los fondos que provienen del presupuesto por resultados (PpR), no son utilizados en las actividades de las estrategias de tuberculosis y VIH, ya que las autoridades de los establecimientos destinan estos recursos a otros gastos.

“*[...] porque las autoridades utilizan el presupuesto para otras cosas. La lucha es constante para tratar de proteger los fondos que tenemos, algunos nos apoyan y otros no, su visión es más asistencialista [...]”* (IQT_01_III)

En el Perú, la escasez de personal de salud se debe no solo a la falta de profesionales, sino también a su distribución desigual. Esto conlleva a que los profesionales de salud tengan que repartirse entre labores administrativas y asistenciales. En el caso de las estrategias de TB y VIH, el personal no solo se encarga de la atención diaria de los pacientes, sino también de la programación, gestión y reporte de las actividades. A esto se suma la alta rotación del personal por falta de incentivos laborales, lo cual repercute en todas las actividades de ambas estrategias.

“*[...] tenemos que hacer labor asistencial y administrativa, y acá hay gente que también lleva microrred además del centro, entonces es como dos responsabilidades”*. (SJL_01_IIa)

La falta de personal está ligada también al financiamiento insuficiente. Además, el recurso humano carece de capacitación y actualización, sobre todo en el primer nivel.

### Componente cuantitativo

**FIGURA 3.**
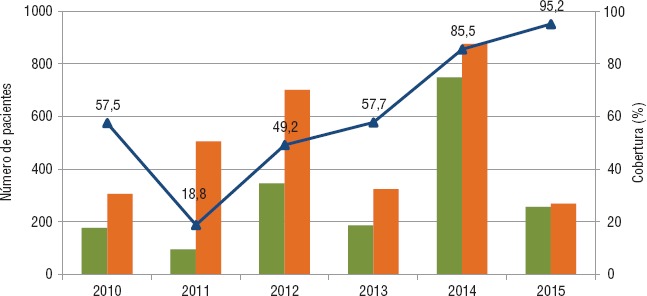
Cobertura de tamizaje para VIH en pacientes con TB en todos los establecimientos de salud del estudio, 2010–2015

VIH, virus de la inmunodeficiencia humana; TB, tuberculosis.

La cobertura de tamizaje para VIH en pacientes con tuberculosis se incrementó de manera progresiva de 18,8% en 2011 a 95,2% en 2015 (figura 3). No se encontró información sistematizada en los registros de VIH sobre el descarte del diagnóstico de tuberculosis.

**FIGURA 4.**
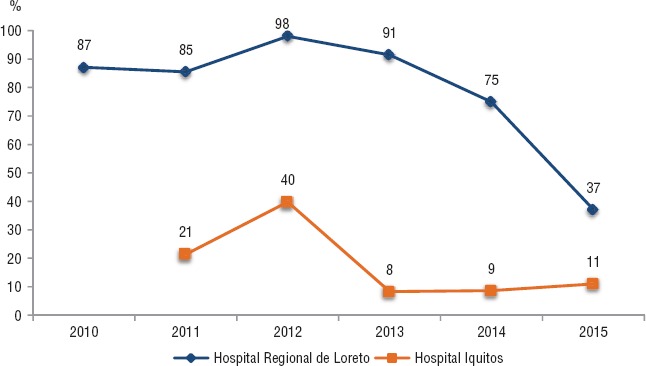
Porcentaje de pacientes con VIH que iniciaron TPI por año, Iquitos, 2010–2015

VIH, virus de la inmunodeficiencia humana; TPI, terapia preventiva con isoniazida.

La cobertura de la terapia preventiva con isoniazida (TPI) en el Hospital Regional fue mayor al 85% entre los años 2010 y2013 y disminuyó a 37% en 2015. En el hospital Iquitos, la cobertura de TPI disminuyó de 40% en 2012 a 11% en 2015 (figura 4). No se encontró información integrada con la estrategia de tuberculosis sobre los pacientes coinfectados que reciben TARV, ya que suelen atenderse en diferentes establecimientos de salud.

De los 266 pacientes con coinfección por TB/VIH (225 en Iquitos y 41 en Lima) entre 2010 y 2015, el Hospital de Iquitos reportó un caso de tuberculosis multidrogorresistente (TB-MDR) en 2012, mientras que el Hospital San Juan de Lurigancho tuvo ocho casos. Además, se registraron casos de tuberculosis monorresistente: dos en el Hospital Regional de Loreto y dos en el Puesto de Salud Sagrada Familia. No se registraron casos de tuberculosis extremadamente resistente.

En total hubo 52 fallecidos entre los casos de coinfección por TB/VIH, con una proporción de 20%, desde 2010 a 2015. Esta proporción fue mayor en Lima que en Iquitos (24% frente a 19%).

## DISCUSIÓN

El presente estudio permitió identificar las barreras programáticas que afectan el acceso a la atención integral de las personas afectadas por la coinfección TB/VIH en siete establecimientos de salud de Lima e Iquitos, Perú. Entre ellas se destacan las dificultades para la coordinación entre las estrategias de tuberculosis y VIH, la fragmentación de la atención en diferentes establecimientos de salud, la ausencia de documentos normativos para el manejo de esta condición y la falta de un sistema de información integrado. Otras barreras son el insuficiente financiamiento para las actividades y la escasez de personal de salud o su falta de capacitación. Se ha evidenciado además que algunos indicadores, como el tamizaje para VIH en pacientes con tuberculosis, han mejorado de manera notable. Sin embargo, la cobertura de isoniazida en pacientes con VIH aún se encuentra muy por debajo de la meta nacional, que es 100%.

La diferencia más importante de los indicadores evaluados en los establecimientos de salud de Lima e Iquitos son las coberturas de TPI. Esto se puede explicar por una falta de implementación de la normativa vigente o debilidades en el suministro de medicamentos en la región selva, entre otras posibles explicaciones que pueden ser profundizadas en otro estudio.

La colaboración entre los programas de tuberculosis y VIH ha sido analizada en varios estudios, en los cuales se encontró que la falta de integración en los distintos niveles es un problema común a varios contextos (12, 16–19). Similar a nuestro estudio, otras investigaciones han encontrado que las consecuencias de esta falta de integración son la dificultad para el seguimiento, la pérdida de casos, la fragmentación de la atención y el incremento de oportunidades perdidas (16, 19). Además, se ha encontrado que estas barreras tienen efectos sobre indicadores como la cobertura de TPI en pacientes con VIH y la alta mortalidad en pacientes con coinfección, además de la falta de datos para la construcción de indicadores como cobertura de TARV en pacientes con tuberculosis.

La falta de financiamiento y de recursos humanos son otras dos barreras importantes que suelen estar presentes en el sistema de salud peruano (20, 21). Al igual que en nuestro estudio, estas barreras limitan la integración de las actividades conjuntas de tuberculosis y VIH (22, 23). Asimismo, la falta de capacitación del personal es un factor clave, ya que contribuiría con un mejor manejo de la coinfección TB/VIH, así como de los mecanismos disponibles para agilizar los procesos en favor de los pacientes. El documento técnico deberá proporcionar un marco legal que genere cambios en los procedimientos administrativos y que a su vez faciliten la formación de recursos humanos y la sostenibilidad de las intervenciones sanitarias.

En el presente estudio no se han analizado las barreras desde el punto de vista del paciente. Otros estudios han encontrado que factores personales como el estilo de vida, problemas socioeconómicos, el soporte social, falta de comprensión del tratamiento, el estigma y discriminación o el consumo de alcohol y drogas afectan el tratamiento de la coinfección por TB/VIH (24–27). Si bien estos factores son importantes, son difíciles de solucionar desde el punto de vista programático, ya que requieren incluso un enfoque multisectorial. Por otra parte, se ha encontrado que un mejor desempeño del sistema de salud (medido por el contexto, la integración, los servicios de soporte, los recursos humanos y la continuidad y calidad del servicio) está relacionado con mejores resultados del tratamiento (cura o cumplimiento de la terapia) en pacientes con coinfección por TB/VIH (28).

La limitación principal de este estudio es el posible sesgo de deseabilidad social, sobre todo por parte de los pacientes, dado que las entrevistas fueron realizadas en los establecimientos de salud. También pudo haber sesgo de selección de participantes, debido a que se entrevistó a aquellos que estaban disponibles al momento de la visita. Sin embargo, la información obtenida es consistente según el tipo de actor clave entrevistado, lo cual nos da confianza de haber explorado las barreras de manera adecuada. Sobre el componente cuantitativo, las limitaciones tienen que ver con la fuente de información, ya que los registros pueden subnotificar los casos o las actividades. Se espera que este sesgo sea similar entre establecimientos y que, por lo tanto, no afecte las comparaciones. No obstante estas limitaciones, este estudio aporta información que permitirá a los tomadores de decisiones identificar las debilidades, desde el punto de vista del sistema, de la atención de la coinfección por TB/VIH.

Por último, en este estudio se ha identificado como una barrera la falta de un documento técnico para la atención de la coinfección por TB/VIH. Es por ello que, durante el desarrollo de esta investigación, se elaboró un documento que permite la estandarización de los procesos de atención integral del paciente con coinfección por TB/VIH, abordando las principales barreras encontradas. Además, se organizó una reunión entre los equipos de tuberculosis y VIH del nivel central y de algunos establecimientos de salud de Lima para exponer algunos aspectos contenidos en este documento técnico. Sin embargo, para los siguientes pasos de la validación y posterior implementación, se deberá involucrar a todos los actores; de lo contrario, correría el riesgo de no ser tomado en cuenta en el nivel operativo (29).

## CONCLUSIONES

Se encontró una débil coordinación entre las estrategias sanitarias de VIH y TB en establecimientos de salud incluidos en el estudio. El manejo de la coinfección por TB/VIH está fragmentado, lo que dificulta la atención integral del paciente. Estos resultados muestran la necesidad de contar con políticas y normativas específicas para el problema de la coinfección por TB/VIH en el país, por lo que se ha elaborado un documento técnico conjunto sobre TB/VIH. Este documento establece intervenciones sanitarias y procedimientos administrativos en los servicios de salud a nivel nacional y busca disminuir la incidencia, la morbilidad y la mortalidad de las personas afectadas por la TB y el VIH en el Perú.

### Agradecimientos.

Los autores desean agradecer a Ariel Bardach, Sebastián García y Fernando Rubinstein, del Instituto de Efectividad Clínica y Sanitaria, por sus valiosas contribuciones durante la elaboración del proyecto; a Miluska Carrasco y Hellen Palma por el desarrollo del trabajo de campo. Agradecen también a las autoridades y trabajadores de la Dirección Regional de Salud de Loreto (Hermann Silva y Ceci Ríos) y de la Red de Salud de San Juan de Lurigancho (Nancy Zerpa, Corina Vásquez y Flor Domínguez) por facilitarnos la realización de esta investigación. Finalmente, los autores agradecen a los participantes de este estudio por la valiosa información que han brindado.

### Financiamiento.

Este trabajo fue financiado por la Alianza para la Investigación en Políticas y Sistemas de Salud (AHPSR), de la Organización Mundial de la Salud (OMS). La Organización Panamericana de la Salud brindó cooperación técnica para el desarrollo de este proyecto.

### Declaración.

Las opiniones expresadas en este manuscrito son responsabilidad del autor y no reflejan necesariamente los criterios ni la política de la *RPSP/PAJPH* y/o de la OPS.
